# Making sense of change after Intensive Trauma Treatment: a mixed-methods study into adolescents’ experience of efficacy

**DOI:** 10.1186/s13034-024-00781-5

**Published:** 2024-07-25

**Authors:** Elisa van Ee, Dani de Beijer, Desirée Florisson, Fenna Geuskens

**Affiliations:** 1 Psychotraumacentrum Zuid-Nederland, Bethaniëstraat 10, Den Bosch, The Netherlands; 2 Behavioural Science Institute, Radboud University, Thomas van Aquinostraat 4, Nijmegen, The Netherlands; 3 Herlaarhof, Boxtelseweg 32, Vught, The Netherlands

**Keywords:** PTSD, Trauma treatment, Adolescence, Social support, Identity

## Abstract

**Background:**

While evidence-based interventions are effective for children with post-traumatic stress disorder (PTSD), some adolescents may not respond sufficiently. Intensive trauma treatment (ITT) has shown promise for adults, but research on its efficacy for adolescents is limited. This study therefore aimed to explore the efficacy and subjective experience of change in adolescents participating in ITT.

**Methods:**

The present study employed a mixed-methods approach among a sample of adolescents with PTSD (*N* = 22; 90.1% female, age *M* = 17.0, *SD* = 1.72*)* who participated in an ITT program. Clinical data and narratives were combined to assess treatment efficacy and subjective experiences of change.

**Results:**

Quantitative analysis revealed a significant reduction in PTSD symptoms post-ITT, aligning with prior research. Qualitative analysis highlighted themes such as negative thoughts impacting treatment success, the importance of social support, and identity-related struggles.

**Conclusions:**

The study contributes to understanding ITT efficacy and emphasizes the need for developmental sensitivity, systemic interventions, and continued research to enhance PTSD treatment for adolescents.

## Introduction

Adverse childhood experiences (ACEs) constitute a considerable public health issue, exerting enduring effects on the psychological, emotional, and social well-being of those affected [1, 2]. Epidemiological studies reveal the pervasive nature of exposure to various traumatic events among youth globally, including physical or sexual violence, mass conflict, serious accidents, natural disasters, domestic abuse, and neglect [3–6]. General population estimates indicate that one to two-thirds of children undergo potentially traumatizing events before the age of 16 [7, 8]. Approximately 16% of these exposed children and adolescents develop post-traumatic stress disorder (PTSD; [9]), with heightened risks of comorbid depressive symptomatology, addiction, and/or developmental problems [10–12].

In recent years, there has been an increased recognition of the need for effective trauma-focused interventions tailored specifically to children and young adolescents. Previous research provides substantial evidence for the efficacy of various evidence-based interventions, including Trauma-Focused Cognitive Behavioral Therapy (TF-CBT; [13, 14]) and Eye Movement Desensitization and Reprocessing (EMDR; [15, 16]). However, for a subset of youth, particularly those with more complex, interpersonal trauma and/or comorbid psychopathology, evidence-based guideline treatments may not always be as effective and accessible. Not all children and adolescents exhibit sufficient improvement, and dropout rates from trauma-focused treatment remain concerning [17–19].

A potential solution to these challenges involves enhancing current guideline treatments by integrating treatments and condensing sessions into a shorter timeframe, creating an intensive trauma treatment (ITT) program [20]. Intensive trauma treatment targeting adults has shown promising results in reducing PTSD symptoms and overcoming avoidance behaviors [21–24]. Despite being considered a safe, accessible treatment for adults with PTSD [25], a significant gap remains in our understanding of the effectiveness of ITT for children and adolescents. Nevertheless, research results indicate that ITT for adolescents can potentially be safe and effective, but more research is necessary [26–28].

This study aims to contribute to the existing literature by investigating the efficacy of an ITT program for adolescents using a mixed-methods approach. What makes this study unique is the combination of analyzing clinical data and youths’ narratives. This approach is particularly suitable for understanding efficacy beyond clinical outcomes of PTSD symptomatology, hereby providing unique insights into the subjective experience of adolescents participating in ITT, and more specifically, how adolescents experience and make sense of changes in symptomatology and daily functioning. The study will extend the existing knowledge of traumatized youths’ experiences of receiving TF-CBT [29, 30]. In terms of PTSD symptomatology, the hypothesis posited is that participation in ITT will reduce PTSD symptoms, aligning with prior results from studies on adult ITT programs and the first studies targeting ITT programs for youth.

## Methods

### Design and setting

The study adopted a retrospective cohort design, utilizing a mixed-methods approach to investigate the efficacy of ITT. The quantitative component encompassed pre- and post-treatment assessments of PTSD symptomatology, while the qualitative component involved narratives written by participating youth during treatment. The research transpired at Herlaarhof, a prominent community mental health center in the Netherlands specializing in severe psychiatric disorders among children and adolescents. Renowned for its expertise in PTSD and related disorders, Herlaarhof introduced a tailored 4-week ITT program in 2020. This program specifically targeted youth exposed to multiple traumas or those who did not benefit adequately from prior interventions. The ITT program amalgamated evidence-based therapies such as psycho-education, EMDR, imaginary exposure, and in-vivo exposure. Supplementary elements like family interventions, creative therapy, psychomotor therapy, and writing therapy were also integrated. The program adopted a therapist rotation model. For a more detailed description of the program see [31].

### Participants

A total of 39 adolescents, aged between 12 and 21, participated in ITT at Herlaarhof from August 2020 to October 2022. To qualify for ITT, participants needed to exhibit PTSD symptomatology and express motivation for short-term intensive treatment. ITT was not recommended in cases of immediate danger, where the source of trauma was ongoing. The study included only youth with a PTSD classifying diagnosis. Among the initial sample of 39 adolescents, 17 participants were excluded due to incomplete data (*n* = 15), absence of a PTSD diagnosis (*n* = 1), and unfinished treatment (*n* = 1). Consequently, the final quantitative analysis involved a sample of 22 participants (90.1% female, Mage = 17.0, SD = 1.72). Consent for the analysis of their narratives was asked of those 22 participants with half of the sample providing consent, resulting in a final qualitative analysis sample of 11 youth.

### Measures

#### CAPS-CA-5

A Dutch translation of the Clinician-Administered PTSD Scale for DSM-5 – Child/Adolescent Version (CAPS-CA; [32]) was utilized to establish PTSD classification and symptom severity. The CAPS-CA-5 is a validated semi-structured diagnostic interview that assesses PTSD symptoms following criteria of the Diagnostic and Statistical Manual of Mental Disorders (DSM-5; [33]). The interview includes a life-event checklist and 30 items targeting symptom frequency and severity. Results are summed to calculate total symptom severity (combining frequency and severity) and individual symptom cluster severity scores. PTSD classification is determined by assessing the “presence” or “absence” of each symptom, following the DSM-5 diagnostic rule. A symptom is considered present if the corresponding item severity score is rated 2 or higher.

#### Narratives

Participants, along with a trauma therapist, composed a short narrative using a provided form as a guide. The narrative covered relevant treatment goals, self-observations during treatment, and reflections on both the retrospective view of their treatment experience and their prospective outlook towards the future. To facilitate writing, probes such as ‘Before ITT I…’, ‘During ITT my goal was…’, ‘I succeeded in…’ and ‘In the future…’ were used. The narrative form is part of Herlaarhof’s clinical ITT program and was not specifically developed for research purposes. It is available upon request.

#### Data collection

Pre- and post-test measures of PTSD classification and symptom severity occurred in the week before and five weeks after treatment, respectively. Youth narratives were collected in the week after treatment. All collected data were considered part of the ITT treatment protocol and were documented in the patient files. Results from CAPS-CA-5 were deemed anonymous data by the local ethical committee; hence, no informed consent was required for its use. Informed consent for the use of narratives was obtained through an information letter and consent form sent to all patients who received ITT. Practitioners also inquired whether patients gave consent to be contacted by the research team.

### Data analysis

Quantitative analysis involved paired samples T-tests to assess differences between pre- and post-test CAPS-CA 5 measures (total symptom severity scores and DSM-5 PTSD symptom cluster severity scores), two-tailed tests were employed, with statistical significance set at *p* < .05, and a chi-square test to assess changes in DSM-5 PTSD diagnosis. Cohen’s d was employed as an index of effect size, with thresholds interpreted as small (0.20), medium (0.50), and large (0.80) [34].

Qualitative analyses were conducted from a critical realist perspective, assuming that there is a reality to be investigated while recognizing that representations of this reality are influenced by the culture, language, and interests of the participant. Inductive thematic analysis, as described by Braun & Clarke [35], was used, involving reading and rereading narrative transcripts, initial pattern identification, line-by-line coding, and checking for patterns of variability and consistency. Interviews were coded by the last author under the supervision of the first author, who is experienced with qualitative analysis. Themes were identified at both the manifest and latent levels, based on the knowledge gained from assessing available trauma literature. Themes were identified by the first author and refined until they formed a coherent pattern with the coded data, using the software program Atlas.ti.

## Results

### Differences between pre- and post-test CAPS-CA 5 measures of total symptom severity and symptom cluster severity

Paired samples t-tests were conducted to examine the disparities in CAPS-CA 5 total symptom severity and symptom cluster severity scores between the pre- and post-test assessments. The results indicated a significant reduction in total symptom severity scores for participants after treatment (M = 51.14, SD = 9.463), compared to before treatment (M = 34.18, SD = 17.933, t(21) = 5.005, *p* < .001). The effect size, measured by Cohen’s d, was d = 1.18, signifying a large effect.

Regarding symptom cluster severity, the findings revealed a significant difference between criterion B scores before treatment (M = 14.27, SD = 3.089) and after treatment (M = 8.27, SD = 6.112, t(21) = 4.767, *p* < .001), criterion C scores before treatment (M = 5.77, SD = 1.510) and after treatment (M = 3.23, SD = 2.409, t(21) = 4.506, *p* < .001), criterion D scores before treatment (M = 18.09, SD = 4.196) and after treatment (M = 12.36, SD = 5.860, t(21) = 4.713, *p* < .001), and criterion E scores before treatment (M = 13.00, SD = 3.491) and after treatment (M = 10.45, SD = 5.829, t(21) = 2.488, *p* = .021). The effect sizes for each criterion, as measured by Cohen’s d, were d = 1.24, d = 1.27, d = 1.12, and d = 0.53, respectively, indicating a large effect size for criterion B, C, and D, and a medium effect size for criterion E.

### Changes in PTSD diagnostic status after treatment

A chi-squared test was employed to assess differences in PTSD diagnostic status based on pre- and post-test CAPS-CA 5 measures. Results demonstrated a significant difference in PTSD diagnostic status after treatment, with 8 out of 22 participants (36.4%) no longer meeting the criteria for PTSD.

#### Themes from narratives

Qualitative analysis of narratives resulted in three themes summarized in Table 1.

**Table 1:** Theme summary table

Theme	Characteristics
Negative thoughts and feelings at the core	Negative thoughts and feelings central in symptoms and treatment expectations.
Sharing diminishes loneliness	Increase in sharing fosters social support and trust in social network
Who am I?	Trauma and treatment shape identity, development continues after treatment

#### Negative thoughts and feelings at the core

Negative thoughts and feelings play a central role in the narratives of youth participating in ITT, influencing their PTSD symptomatology and daily functioning. The narratives delve into how emotions such as self-criticism, self-blame, and mistrust become compelling reasons for seeking ITT. The negative thoughts and feelings often lead them to feel anxious, prompting withdrawal from school and social activities. In contrast to the burden of shame, guilt, and mistrust, they express a desire “to feel normal again,” “to pick up normal life,” and “to enjoy life and have fun again.” While these negative thoughts and feelings act as a motivator for seeking help, they also pose a significant obstacle, instilling doubts in the youths about their ability to successfully complete therapy. The uncertainty about what to expect from treatment intensifies their internal struggles. Questions like “Can I handle it?” and “What if it succeeds, what if it doesn’t succeed?” loom large in their minds. Upon entering therapy, they describe ITT as “intensive,” “difficult,” and “confronting.” The process stirs up a range of emotions, including anger, anxiety, panic, and sadness, challenging their ability to regulate these intense feelings. Despite the emotional upheaval, they undergo a transformative experience, expressing that it was positive to encounter new emotions. Shame and guilt transform into feelings of an unfairness, and they find themselves better equipped to acknowledge, express, and regulate their emotions. However, the introduction of these ‘new’ emotions can also leave adolescents feeling overwhelmed or confused.

#### Sharing diminishes loneliness

Apart from the attitude to “just go for it,” social support emerges as a crucial aspect influencing their decision to start, endure, and complete ITT. Friends, family, fellow patients, and ITT team members are perceived as valuable sources of support. The ability to share, ask questions, the presence of others, the offered distractions, and the opportunity to feel normal contribute to their sense of being supported. “It felt like they took me seriously”. Social support, however, involves a delicate balance. Parents, for example, walk a fine line between offering too much or too little support, which can be perceived as unwanted interference or neglect. Throughout the ITT process, youth gain insights into the significance of their social network, emphasizing the importance of sharing experiences: “the past is not gone, but talking about it helps” and “I feel less lonely when I share my story”. This increase in sharing fosters greater trust in themselves and their social network. Consequently, feeling supported is crucial for youth to actively engage in ITT, and the process of sharing in ITT reinforces their trust in the received support. This is evident in statements like “I share more with my parents” and “My trust in others has increased.”

#### Who am I?

The narratives also highlight a pronounced quest for identity among youth undergoing ITT. While identity exploration is a natural part of their developmental phase, the experience of trauma shapes and complicates this quest. On one hand, the youth express a sense of pride upon completing therapy, realizing their capability to overcome challenges and move beyond avoidance. They assert, “I experienced that I am able to do this, that I can stop avoiding”, “I am a diehard” and “I am more than my trauma.” On the other hand, there is a pervasive sense of confusion about their identity, especially after enduring trauma symptoms for an extended period. Questions such as “Who am I, now that I am not bothered by the trauma symptoms anymore?” underscore their insecurity about the newfound changes. Previously confined by anxiety, they now grapple with new emotions and thoughts, making it challenging to navigate their understanding of self. The struggle to handle these unfamiliar emotions prompts reflections on self-stability. “It was difficult to handle those emotions that I had not experienced before”, “All sorts of new questions came to mind”, “How can I understand myself better?”, “I didn’t feel stable”. It appears as if these adolescents need to forge a new identity for a life beyond trauma. Importantly, most adolescents express a desire to explore this theme further in psychotherapy post-ITT.

## Discussion

To date, limited research has explored the efficacy and applicability of ITT for youth. This study sought to delve into traumatized youths’ experiences of undergoing ITT. As expected, quantitative analyses revealed a statistically significant decrease in PTSD symptoms among participants in the ITT program. This reduction was evident in both the overall symptom severity and the severity of individual DSM-5 symptom clusters. The decline in PTSD symptoms resulted in a loss of PTSD classification among 36,4% of the sample. These results align with previous research on both adults [20, 23, 36] and adolescents [26–28]. The observed percentage of classifying diagnosis loss post-treatment was comparatively on the lower end compared to earlier studies [37]; however, it aligns with existing research suggesting a continued loss of PTSD classification over follow-up periods [28].

Various factors may contribute to differences in study outcomes. One relevant factor, for example, is the structure and content of ITT programs. In the study of Hendriks et al., ITT primarily involved exposure as the main treatment [28], whereas in the current study and in the study of van Pelt et al., exposure and EMDR were combined [26]. Additionally, factors not directly related to trauma treatment should be considered when interpreting differences in study results. For instance, Hoogsteder et al., identified age as a moderator in their meta-analysis on the treatment effect of EMDR and TF-CBT in reducing trauma symptoms and externalizing behavior problems in youth, noting that younger participants tend to have better outcomes [38]. Differences in mean age between the current study (17.0) and previous ITT studies (Hendriks et al., (15.9) [28], van Pelt et al., (16.1) [26]) are therefore important to consider. Comorbidity is a another relevant factor [39, 40]. In previous studies [26, 28], the mean number of comorbid disorders was more than two, whereas the number of comorbid disorders was not assessed in the current study. Although sex is considered a relevant factor in trauma treatment outcomes [41], it does not explain differences in outcome between the current study and previous studies, as the percentage of females in all studies was remarkable high (over 80%) [26, 28]. Future research should further explore factors such as age, sex, and comorbidity, as well as other variables like therapist rotation [42], number of sessions, and treatment length [43].

Qualitative analyses uncovered themes shedding light on how adolescents perceive changes in PTSD symptoms during and after ITT. In line with studies on TF-CBT [29, 30], one prominent theme was the experience of negative thoughts and emotions at the outset of and during treatment. These narratives illustrated the fear of handling or benefiting from ITT, potentially hindering treatment success. Intense and sometimes confusing emotions during and after treatment were reported, providing insight into why certain PTSD symptoms (e.g. arousal or restlessness) may persist post-treatment, but as confidence in recovery increases, continue to decline over time.

Social support emerged as a significant theme in understanding how adolescents experience and benefit from ITT. Social support is known to play a substantial role in enhancing mental health [44], mitigating the consequences of trauma-induced disorders [45], and promoting treatment success and adherence for both children and adults [46–48]. Indeed, the study results highlight the positive impact of sharing narratives, demonstrating its potential to alleviate feelings of loneliness. This finding aligns with research conducted by Yasinski et al. [49], which emphasized the significance of in-session caregiver behavior as a predictor of clinical outcomes for youth undergoing TF-CBT in various treatment phases. It extends previous research results by emphasizing that youth receive support from a broader social network beyond just their parents, which is crucial because caregiver involvement can present challenges and potentially hinder treatment as well [29, 30]. Future research endeavors can explore different methods and mechanisms through which social support impacts ITT treatment outcomes. Understanding these dynamics can contribute to refining and optimizing ITT interventions, ensuring they comprehensively address the multifaceted needs of adolescents recovering from trauma.

Identity confusion constituted a third significant theme within youth’s narratives. Considering that identity formation is a fundamental developmental milestone of adolescence [50], it is noteworthy that this identity confusion may not necessarily diminish through ITT and may still demand attention post-treatment. Previous studies have demonstrated that childhood trauma can exacerbate identity confusion and have adverse effects on identity formation, leading to lower self-esteem, compromised daily functioning, and a diminished sense of identity [51–53]. Recognizing the relevance and potential impact of identity issues during adolescence, particularly among traumatized youth, ITT programs designed for adolescents could benefit from incorporating trauma-informed interventions that specifically reinforce developmental milestones. Such an approach would acknowledge the challenges youth face in their identity development and provide targeted support throughout the therapeutic process.

The study is subject to several limitations. Firstly, the retrospective design employed lacks a control group, impeding our ability to definitively attribute the observed results to the treatment itself rather than other contributing factors. Secondly, the study’s relatively small sample size, coupled with a lack of gender diversity and underrepresentation of male participants, limits the generalizability of the findings. Despite the small sample size, post hoc power analyses (using G*Power 3 [54]) indicated that the paired samples t-tests assessed in this study had 0.99 power to detect a significant effect. Thirdly, the narratives produced during the study may have been influenced by external factors, including the anticipation of sharing and the close interaction with a therapist during the writing process. These external influences could have prompted participants to introduce, alter, or omit specific details in their narratives, introducing a potential source of bias. Lastly, a potential bias may exist in the subgroup of participants who consented to the analysis of their narrative. A substantial group opted not to provide consent, raising uncertainties about whether the consenting subgroup is an adequate representation. These limitations underscore the need for caution when interpreting the study’s findings.

## Conclusions

Despite its limitations, the current study contributes a novel perspective to the still limited body of research on ITT for adolescents. The findings not only underscore the efficacy of ITT in reducing PTSD symptomatology but also offer unique insights into the lived experiences of youth undergoing ITT. Overall, these experiences highlight the potential advantages of systemic and developmentally sensitive interventions in ITT, acknowledging the distinct challenges and vulnerabilities faced by adolescents in the aftermath of trauma. In essence, the study emphasizes the significance of going beyond mere clinical symptom change. Recognizing the importance of a holistic approach that considers both clinical outcomes and the lived experiences of individuals is crucial for advancing the field and improving the overall well-being of traumatized youth.

## Data Availability

The datasets generated and/or analysed during the current study are not publicly available due to privacy but quantitative data are available from the corresponding author on reasonable request.

## Abbreviations

PTSD: Post-traumatic stress disorder
ITT: Intensive Trauma Treatment
ACE: Adverse childhood experience
TF-CBT: Trauma-Focused Cognitive Behavioral Therapy
EMDR: Eye Movement Desensitization and Reprocessing
DSM-5: Diagnostic and Statistical Manual of Mental Disorders, Fifth Edition
CAPS-CA-5: Clinician-Administered PTSD Scale for DSM-5–Child/Adolescent Version
